# Results of a collaborative study on DNA identification of aged bone samples

**DOI:** 10.3325/cmj.2017.58.203

**Published:** 2017-06

**Authors:** Daniel Vanek, Bruce Budowle, Jitka Dubska-Votrubova, Angie Ambers, Jan Frolik, Martin Pospisek, Ahmed Anwar Al Afeefi, Khalid Ismaeil Al Hosani, Marie Allen, Khudooma Saeed Al Naimi, Dina Al Salafi, Wafa Ali Rashid Al Tayyari, Wendy Arguetaa, Michel Bottinelli, Magdalena M. Bus, Jan Cemper-Kiesslich, Olivier Cepil, Greet De Cock, Stijn Desmyter, Hamid El Amri, Hicham El Ossmani, Ruth Galdies, Sebastian Grün, Francois Guidet, Anna Hoefges, Cristian Bogdan Iancu, Petra Lotz, Alessandro Maresca, Marion Nagy, Jindrich Novotny, Hajar Rachid, Jessica Rothe, Marguerethe Stenersen, Mishel Stephenson, Alain Stevanovitch, Juliane Strien, Denilce R. Sumita, Joanna Vella, Judith Zander

**Affiliations:** 1 Forensic DNA Service, Prague, Czech Republic; 2 Charles University in Prague, 2nd Faculty of Medicine, Prague, Czech Republic; 3 Center for Human Identification, University of North Texas Health Science Center, TX 76107, USA; 4 Center of Excellence in Genomic Medicine Research (CEGMR), King Abdulaziz University, Jeddah, Saudi Arabia; 5 Institute of Archaeology AS CR, Prague; 6 Biologicals, Ricany, Czech Republic; 7 Charles University in Prague, Faculty of Science, Prague, Czech Republic; 8 Forensic Evidence Department, Abu Dhabi Police General Directorate, Ministry of Interior, United Arab Emirates; 9 Dept. of Immunology, Genetics and Pathology, Science for Life Laboratory, Uppsala university, Biomedical centre, Uppsala, Sweden; 10 Sharjah Forensic Laboratory, Sharjah Police H.Q, Ministry of Interior, United Arab Emirates; 11 Forensic Genetics Department, Fundación de Antropología Forense de Guatemala (FAFG), Guatemala, Guatemala; 12 Laboratorio di Diagnostica Molecolare, Gentilino, Switzerland; ^13^University of Salzburg, Interfaculty Department of Legal Medicine, DNA-Laboratory, Salzburg, ^14^Biomnis, Lyon, France; 15 Nationaal Instituut voor Criminalistiek en Criminologie, Brussels, Belgium; 16 Genetic Laboratory of Royal Gendarmerie, Rabat, Morocco; Austria; 17 Laboratory of Molecular Genetics and the Malta BioBank, Department of Phsyiology & Biochemistry, Biomedical Sciences Building, University of Malta, Msida, Malta; 18 Bayerisches Landeskriminalamt, München, Germany; 19 Azur Génétique, Nice, France; 20 National Institute of Legal Medicine “Mina Minovici”, Bucharest Romania; 21 Department of Forensic Genetics, Institute of Legal Medicine and Forensic Sciences, Charite, Universitätsmedizin Berlin, Germany; 22 Division of Forensic Sciences/Forensic Genetics, Norwegian Institute of Public Health, Oslo, Norway; 23 Institut National de Police Scientifique, Laboratoire de Police Scientifique de Marseille, Marseille, France; 24 Institute of Legal Medicine, Jena University Hospital-Friedrich Schiller University Jena, Jena, Germany; 25 Genomic Engenharia Molecular, Săo Paulo, Brazil

## Abstract

**Aim:**

A collaborative exercise with several institutes was organized by the Forensic DNA Service (FDNAS) and the Institute of the Legal Medicine, 2nd Faculty of Medicine, Charles University in Prague, Czech Republic, with the aim to test performance of different laboratories carrying out DNA analysis of relatively old bone samples.

**Methods:**

Eighteen laboratories participating in the collaborative exercise were asked to perform DNA typing of two samples of bone powder. Two bone samples provided by the National Museum and the Institute of Archaelogy in Prague, Czech Republic, came from archeological excavations and were estimated to be approximately 150 and 400 years old. The methods of genetic characterization including autosomal, gonosomal, and mitochondrial markers was selected solely at the discretion of the participating laboratory.

**Results:**

Although the participating laboratories used different extraction and amplification strategies, concordant results were obtained from the relatively intact 150 years old bone sample. Typing was more problematic with the analysis of the 400 years old bone sample due to poorer quality.

**Conclusion:**

The laboratories performing identification DNA analysis of bone and teeth samples should regularly test their ability to correctly perform DNA-based identification on bone samples containing degraded DNA and potential inhibitors and demonstrate that risk of contamination is minimized.

The quality and reliability of DNA typing results produced by research and forensic laboratories are limited by the amount and condition of the samples processed, presence of inhibitors, sample collection and storage until analysis, and the practices of the laboratory. Due to frequently limited quantity and quality of DNA in bone samples, even low levels of cross-contamination can become a serious problem for obtaining reliable results. Thus, special attention must be paid to both the procedures and the interpretation of data. Errors can occur and, therefore, laboratories should test their competence through proficiency tests (internal and/or external) and collaborative exercises (1-4). Aged bone samples are among the most difficult biological samples for DNA-based analyses (5,6), and the laboratory should have adequate testing capabilities to analyze these types of samples. It is not sufficient to rely on the analysis of standard reference materials or typical participation in proficiency tests or collaborative exercises of more ideal sample types. While desirable, human ostheological material is not considered a typical standard reference material and is not readily accessible to serve as a material for proficiency tests (4,7-10). To address this testing deficiency, the organizers of the collaborative exercise described herein obtained sufficient quantities of two old bone samples that could be distributed and analyzed among a number of laboratories. The purpose of the exercise was to determine whether concordant results could be obtained from two common samples in different laboratories that use varied extraction procedures, different commercial short tandem repeat (STR) kits, different in-house mitochondrial DNA (mtDNA) protocols, and different laboratory-specific interpretation guidelines.

## MATERIALS AND METHODS

### Sample preparation

The initial step of the sample preparation was the selection of appropriate samples for the collaborative exercise (CE) according to the following six criteria. First, to avoid potential ethical issues, the bone specimens had to be at least 150 years old archeological material, without any identity link to a known person (11), and already subjected to scientific examination (anthropology, archeology, etc.). Second, only the middle parts of long bones were used as test samples (12). Third, the sample preparation (ie, decontamination and cleaning) had to follow the protocol specified previously (5,13). Fourth, the bone specimens had to be converted to homogenous bone powder using a liquid nitrogen grinding mill (14,15) before distribution. Fifth, the bone samples had to be successfully typed by at least two commercial kits to select samples that are typable before distributing them to participating laboratories. Sixth, to assure the correctness of the results, the bone powder had to be quality control checked for typability and contamination before the dispatch of the samples (3).

The above criteria are based on the previous experience and published work of the organizing laboratory.

### Collaborative exercise design

Participating laboratories obtained two different samples, Sample 1 and Sample 2, which had been successfully analyzed by the organizing laboratory. Sample 1 was approximately 400 years old, with degraded DNA and difficult to type. Sample 2 was approximately 150 years old and well-preserved, with relatively intact DNA suitable for standard typing procedures. The age of the specimens was determined by archeologists based on the burial pattern and artifacts found at the excavation site (16,17). Laboratories received 600 mg (Sample 1) and 150 mg (Sample 2) of bone powder prepared from cuttings from the *compacta* of the respective femurs. The surface of the femurs was cleaned using a rotary sanding tool (Dremel, Racine,WI, USA). Following the removal of surface material, additional 2-3 mm of the bone were ground away to remove potential contaminants. The cleaned fragment of approximately 2 × 8 cm was further cut into smaller pieces sized 3 × 6 mm. The bone fragments were then placed in a 50-mL tube and further cleaned by inversion for 30 seconds in 5% commercial bleach, 5 × inversion for 30 seconds in 30 mL of distilled water, and inversion for 30 seconds in 96% ethanol. The bone fragments were allowed to air dry completely before grinding. The bone powder was prepared by grinding the bone fragments in the presence of liquid nitrogen using the cryogenic mill SPEX Sample Prep 6770 Freezer/Mill (Spex CentriPrep, USA). All batches of bone powder were tested for potential contamination (single DNA profile by MiniFiler amplification), subsequently pooled, and divided in aliquots. The bone cleaning and grinding were performed by the organizing laboratory to minimize the possible variable effects of bleach (18) and temperature (19) on the results of the collaborative exercise. The laboratories were asked to perform DNA analysis of the samples with methods they routinely use for bone samples or to use the suggested extraction and typing protocol (Table 1). The suggested protocol recommended to use 50 mg of bone powder per silica-based DNA extraction as described by Vanek et al (20) or DNA extraction protocol as described in user’s manual of PrepFiler BTA Forensic DNA extraction kit (LifeTechnologies, USA). Participating laboratories provided a table with results and the original fragment analysis sample files (FSA files) with the printouts of the resulting electropherograms (EPGs).

**Table 1:** DNA extraction and amplification chemistries used on old bone samples by the laboratories participating in Collaborative Exercise*

Laboratory code	Laboratory type^†^	DNA extraction chemistry	STR kits (autosomal)	X-STR typing	mtDNA typing
1	Organizing laboratory	A	A1, B	YES	YES
2	Government	B, C	C, D1	NO	NO
3	University	C	A2, B	YES	YES
4	Government	B, D	E2	NO	YES
5	Government	E	F1, F2	NO	NO
6	Government	A, D	NO	NO	YES
7	Government	F	A1, E2	YES	NO
8	Police	E	B, F2, G1, G2	NO	NO
9	Police	E	B, E2	NO	NO
10	Government	D	A2	NO	NO
11	Private	D	NO	NO	YES
12	Private	D	B, E1	NO	NO
13	Government	C	F1, F2	NO	YES
14	Government	G	B, E2	NO	NO
15	University	B	D2, E1	NO	YES
16	Private	D	D3, D4	NO	NO
17	Police	A	B, E2	NO	NO
18	Police	C	B, E2	NO	NO
19	University	D	NO	NO	YES

*Abbreviations: STR – short tandem repeat; mtDNA – mitochondrial DNA; DNA extraction chemistry codes – A: BTA Prepfiler (Life Technologies, USA), B: Phenol/chloroform, C: EZ1 DNA Investigator kit (Qiagen, Germany), D: QIAamp/DNeasy kit (Qiagen, Germany), E: Maxwell 16 (Promega Corporation, USA), F: QuickGene (FujiFilm, Japan), G: MagAttract DNA Mini M48 Kit (Qiagen, Germany). STR kit codes – A1: NGM, A2: NGM Select (Life Technologies, USA), B: MiniFiler (Life Technologies, USA), C: Investigator ESSplex SE (Qiagen, Germany), D1: PowerPlex 16, D2: PowerPlex 18D, D3: PowerPlex 16HS, D4: PowerPlex Fusion (Promega Corporation, USA), E1: Identifiler, E2: Identifiler Plus (Life Technologies, USA), F1: PowerPlex ESI17, F2: PowerPlex ESX17 (Promega Corporation, USA), G1: MPX-5, G2: AUX-1 (Serac, Germany).

†All laboratories submitting results for X chromosome STRs used Investigator® *Argus X*-12 (Qiagen, Germany).

## RESULTS

### DNA quantitation and STR typing

The results of DNA quantitation varied substantially across laboratories (Table 2). Five out of 19 participating laboratories did not quantify DNA extracts prior to polymerase chain reaction (PCR).

**Table 2:** DNA quantitation chemistries used for bone sample analysis by the laboratories participating in Collaborative Exercise*

Laboratory code	DNA quantitation chemistry	Sample 1 (ng/μL)^†^	Sample 2 (ng/μL)^†^
**1**	LM	0.00173	0.078
2	QQ	0	0.0316
3	QQ	0	0.046
4	QA	0.00616	0.0415
5	PP	0.01065	0.01411
6	LM	0.00001347, 0.000002232	0.00175, 0.07618, 0.10835, 0.0126, 0.1345
6	QU	1.07, 1.27, 0.808	1.29, 12.6, 1.7
7	QDA	0.00126	0.0177
8	QA	0.089, 0.019, 0.005	0.084
9	QA	0	0.2
10	QU	0	10
11	NA	x	x
12	QA	0.0135, 0.018	0.868
13	NA	x	x
14	NA	x	x
15	LM	0.06	0.8
16	NA	x	x
17	QDA	0	0.107
18	QDA	0.00201	0.121
19	NA	x	x

*Abbreviations: DNA quantitation chemistries codes – QQ: Quantiplex (Qiagen, Germany), QA: Quantifiler Human DNA Quantification Kit (Life Technologie, USA.), QDA: Quantifiler Duo DNA Quantification Kit (Life Technologies, USA), LM: laboratory made RT-PCR quantitation system, PP: Plexor HY System (Promega Corporation, USA), QU: Qubit (Life Technologies, USA), x: no quantitation.

^†^More numbers means quantitation performed several times.

The STR types for the two bone samples were obtained by the CE organizers (Tables 3a and 3b). While the true types of these bones were unknown *a priori*, these STR results were assumed correct for the purpose of the collaboration. The only exceptions were the STR loci D5S818 and SE33 for Sample 1, and D5S818, TPOX, SE33, Penta D, and Penta E for Sample 2, where the consensus results based on the majority rule were considered the correct types. The TPOX, Penta D, and Penta E loci were not evaluated for Sample 1. The negative controls provided by the participating laboratories did not show any evidence of contamination. A situation where a laboratory failed to produce results for a specific STR locus or if only 1 allele at a heterozygous locus was obtained was not considered an error, but a partial result for the purpose of the study. Results with concordant calls, either complete or partial at a locus, with those of the CE organizing laboratory were considered correct (Tables 4a and 4b). Fifteen laboratories submitted results for autosomal STRs. Three laboratories obtained full and concordant profiles for Sample 1, while 13 of 14 laboratories obtained full and concordant profiles for Sample 2. The success rates for autosomal STR typing ranged from 4.5% to 100% for Sample 1 and from 77.3% to 100% for Sample 2. Success was based on the total number of loci a laboratory assayed in this study. Therefore, the percentage of success might be affected by use of more loci. For example, laboratories 17 and 18 both used the MiniFiler kit, which contains only 9 STRs, and had 100% success. Most other laboratories typed more loci and tended to have a lower percentage of success.

**Table 3a:** Results of autosomal short tandem repeat (STR) typing of Sample 1 by the laboratories participating in Collaborative Exercise

Laboratory code	D8S1179	D21S11	D3S1358	THO1	D16S539	D2S1338	D19S433	vWA	D18S51	AME	FGA	D10S1248	D22S1045	D2S441	D1S1656	D12S391	D7S820	CSF1PO	D13S317	TPOX	D5S818	SE33	penta D	penta E
1	8,14	30.2,30.2	16,17	9.3,9.3	9,10	18,20	14, 14.2	14,19	14,18	X,X	21,22	13,15	15,15	11,11	15,16	20,22*	8,10	12,12	11,12	NA^†^	10*	14, 21.2*	NA^†^	NA^†^
2	x^‡^	30.2,30.2	x^‡^	9.3,9.3	x^‡^	x^‡^	x^‡^	14^§^	14,18	X,X	x^‡^	13^§^	x^‡^	11,11	x^‡^	x^‡^	x^‡^	9,10^II^	x^‡^	x^‡^	x^‡^	x^‡^	x^‡^	x^‡^
3	x^‡^	30.2,30.2	16,17	x^‡^	9^§^	18,20	14^§^	11,13,19^II^	14,18	X,X	21,22	13^§^	15,15	11,11	x^‡^	x^‡^	x^‡^	x^‡^	11,12	NA^†^	NA^†^	14^§^	NA^†^	NA^†^
4	8,14	x^‡^	16,17	x^‡^	x^‡^	x^‡^	14,14.2,15^II^	x^‡^	x^‡^	X,X	x^‡^	NA^†^	NA^†^	NA^†^	NA^†^	NA^†^	x^‡^	x^‡^	8^II^	8	10	NA^†^	NA^†^	NA^†^
5	8,14	30.2,30.2	16,17	9.3,9.3	9,10	20^§^	14,14.2	14,19	14,18	X,X	21,22	13,15	15, 15	11,11	16^§^	20^§^	NA^†^	NA^†^	NA^†^	NA^†^	NA^†^	21.2^§^	NA^†^	NA^†^
8	8,14	30.2,30.2	x^‡^	9.3,9.3	9^§^	18,20	x^‡^	14,19	x^‡^	X,X	x^‡^	13,15	15,15	11,11	15,16	20,22	NA^†^	NA^†^	NA^†^	NA^†^	NA^†^	14^§^	NA^†^	NA^†^
9	x^‡^	x^‡^	x^‡^	x^‡^	10^§^	20^§^	x^‡^	x^‡^	10,11.2,18^II^	X,Y^II^	21^§^	NA^†^	NA^†^	NA^†^	NA^†^	NA^†^	x^‡^	12,12	11,12	x^‡^	x^‡^	NA^†^	NA^†^	NA^†^
10	x^‡^	x^‡^	x^‡^	x^‡^	x^‡^	x^‡^	x^‡^	x^‡^	x^‡^	x^‡^	x^‡^	x^‡^	x^‡^	x^‡^	x^‡^	x^‡^	NA^†^	NA^†^	NA^†^	NA^†^	NA^†^	x^‡^	NA^†^	NA^†^
12	8,14	30.2,30.2	16,17	9.3, 9,3	9,10	18,20	x^‡^	x^‡^	14,18	X,X	21,22	NA^†^	NA^†^	NA^†^	NA^†^	NA^†^	8,10	12,12	11,12	x^‡^	x^‡^	NA^†^	NA^†^	NA^†^
13	8,14	30.2,34.2^II^	16,17	9.3,9.3	9,10	18,20	14,14.2,15^II^	14,17,19^II^	14,18,21^II^	X,X	21,22	13,15	15,15	11,11	15,16	20,22	NA^†^	NA^†^	NA^†^	NA^†^	NA^†^	12,14,21.2^II^	NA^†^	NA^†^
14	x^‡^	30.2, 30.2	17^§^	x^‡^	9,10	18,20	14.2^§^	x^‡^	14^§^	X,X	21,22	NA^†^	NA^†^	NA^†^	NA^†^	NA^†^	8^§^	12,12	11,12	x^‡^	10	NA^†^	NA^†^	NA^†^
15	x^‡^	x^‡^	x^‡^	x^‡^	x^‡^	x^‡^	x^‡^	x^‡^	x^‡^	x^‡^	x^‡^	NA^†^	NA^†^	NA^†^	NA^†^	NA^†^	x^‡^	x^‡^	x^‡^	x^‡^	x^‡^	NA^†^	x^‡^	x^‡^
16	8^§^	x^‡^	16^§^	9.3,9.3	x^‡^	x^‡^	x^‡^	x^‡^	x^‡^	X,X	x^‡^	x^‡^	x^‡^	x^‡^	x^‡^	x^‡^	x^‡^	x^‡^	11^§^	x^‡^	10	NA^†^	x^‡^	x^‡^
17	x^‡^	30.2,30.2	x^‡^	x^‡^	9,10	18,20	x^‡^	x^‡^	14,18	X,X	21,22	NA^†^	NA^†^	NA^†^	NA^†^	NA^†^	8,10	12,12	11,12	x^‡^	x^‡^	NA^†^	NA^†^	NA^†^
18	NA^†^	30.2,30.2	NA^†^	NA^†^	9,10	18,20	NA^†^	NA^†^	14,18	X,X	21,22	NA^†^	NA^†^	NA^†^	NA^†^	NA^†^	8,10	12,12	11,12	NA^†^	NA^†^	NA^†^	NA^†^	NA^†^

*Consensus profile

†NA – not contained in the kit used.

‡x – no results obtained.

§Missing one allele at heterozygous locus.

IIWrong allele.

**Table 3b:** Results of autosomal short tandem repeat (STR) typing of Sample 2 the laboratories participating in Collaborative Exercise

Laboratory code	NGM STRs																MiniFiler STRs									Additional loci				
D8S1179	D21S11	D3S1358	THO1	D16S539	D2S1338	D19S433	vWA	D18S51	AME	FGA	D10S1248	D22S1045	D2S441	D1S1656	D12S391	D21S11*	D7S820	CSF1PO	D13S317	D16S539*	D2S1338*	D18S51*	AME*	FGA*	TPOX	D5S818	SE33	Penta E	Penta D
1	11,14	32,33.2	15,15	6,9.3	10,11	22,25	13,14	15,20	13,16	X,X	19,20	15,16	15,15	11,14	16,17	20,23	32,33.2	12,13	11,12	11,14	10,11	22,25	13,16	X,X	19,20	8,11^†^	12,13^†^	17,22^†^	10,16^†^	11,11^†^
2	11,14	32,33.2	15,15	6,9.3	10,11	22,25	13,14	15,20	13,16	X,X	19,20	15,16	15,15	11,14	16,17	20,23	32,33.2	12,13	11,12	11,14	10,11	NA^‡‡^	13,16	X,X	19,20	8,11	12,13	17,22	10,16	11,11
3	11,14	32,33.2	15,15	6,9.3	10,11	22,25	13,14	15,20	13,16	X,X	19,20	15,16	15,15	11,14	16,17	20,23	NA^‡‡^	NA^‡^	NA^‡^	NA^‡^	NA^‡^	NA^‡^	NA^‡^	NA^‡^	NA^‡^	NA^‡^	NA^‡^	17,22	NA^‡^	NA^‡^
4	11,14	32,33.2	15,15	6,9.3	10,11	22,25	13,14	15,20	13,16	X,X	19,20	NA^‡^	NA^‡^	NA^‡^	NA^‡^	NA^‡^	NA^‡^	12,13	11,12	11,14	NA^‡^	NA^‡^	NA^‡^	X,X	NA^‡^	8,11	12,13	NA^‡^	NA^‡^	NA^‡^
5	11,14	32,33.2	15,15	6,9.3	10,11	22,25	13,14	15,20	13,16	X,X	19,20	15,16	15,15	11,14	16,17	20,23	32,33.2	NA^‡^	NA^‡^	NA^‡^	10,11	22,25	13,16	X,X	19,20	NA^‡^	NA^‡^	17,22	NA^‡^	NA^‡^
7	11,14	32,33.2	15,15	6,9.3	10,11	22,25	13,14	15,20	13,16	X,X	19,20	15,16	15,15	11,14	16,17	20,23	32,33.2	12,13	11,12	11,14	10,11	22,25	13,16	X,X	19,20	8,11	12,13	NA^‡^	NA^‡^	NA^‡^
8	11,14	32,33.2	15,15	6,9.3	10,11	22,25	13,14	15,20	13,16	X,X	19,20	15,16	15,15	11,14	16,17	20,23	NA^‡^	NA^‡^	NA^‡^	NA^‡^	NA^‡^	NA^‡^	NA^‡^	X,X	NA^‡^	NA^‡^	NA^‡^	17,22	NA^‡^	NA^‡^
9	11,14	x^§^	15,15	6,9.3	10,11	x^§^	13,14	15^II^	13^II^	X,X	x^§^	NA^‡^	NA^‡^	NA^‡^	NA^‡^	NA^‡^	x^§^	12,13	11,12	11,14	10,11	22,25	13,16	X,X	x^§^	11^II^	12,13	NA^‡^	NA^‡^	NA^‡^
10	11,14	32,33.2	15,15	6,9.3	10,11	22,25	13,14	15,20	13,16	X,X	19,20	15,16	15,15	11,14	16,17	20,23	NA^‡^	NA^‡^	NA^‡^	NA^‡^	NA^‡^	NA^‡^	NA^‡^	X,X	NA^‡^	NA^‡^	NA^‡^	17,22	NA^‡^	NA^‡^
12	11,14	32,33.2	15,15	6,9.3	0,10^II^	22,25	13,14	15,20	13,16	X,X	19,20	NA^‡^	NA^‡^	NA^‡^	NA^‡^	NA^‡^	32,33.2	12,13	11,12	11,14	10,11	22,25	13,16	X,X	19,20	8,11	12,13	NA^‡^	NA^‡^	NA^‡^
13	11,14	32,33.2	15,15	6,9.3	10,11	22,25	13,14	15,20	13,16	X,X	19,20	15,16	15,15	11,14	16,17	20,23	NA^‡^	NA^‡^	NA^‡^	NA^‡^	NA^‡^	NA^‡^	NA^‡^	NA^‡^	NA^‡^	NA^‡^	NA^‡^	17,22	NA^‡^	NA^‡^
14	11,14	32,33.2	15,15	6,9.3	10,11	22,25	13,14	15,20	13,16	X,X	19,20	NA^‡^	NA^‡^	NA^‡^	NA^‡^	NA^‡^	32,33.2	12,13	11,12	11,14	10,11	22,25	13,16	X,X	19,20	8,11	12,13	NA^‡^	NA^‡^	NA^‡^
15	11,14	32,33.2	15,15	6,9.3	10,11	22,25	13,14	15,20	13,16	X,X	19,20	NA^‡^	NA^‡^	NA^‡^	NA^‡^	NA^‡^	32,33.2	12,13	11,12	11,14	10,11	22,25	13,16	X,X	19,20	8,11	12,13	NA^‡^	10,16	11,11
16	11,14	32,33.2	15,15	6,9.3	10,11	NA^‡^	NA^‡^	15,20	13,16	X,X	19,20	NA^‡^	NA^‡^	NA^‡^	NA^‡^	NA^‡^	32,33.2	12,13	11,12	11,14	10,11	…	13,16	X,X	19,20	8,11	12,13	NA^‡^	10,16	11,11
17	11,14	32,33.2	15,15	6,9.3	10,11	22,25	13,14	15,20	13,16	X,X	19,20	NA^‡^	NA^‡^	NA^‡^	NA^‡^	NA^‡^	32,33.2	12,13	11,12	11,14	10,11	22,25	13,16	X,X	19,20	8,11	12,13	NA^‡^	NA^‡^	NA^‡^
18	11,14	32,33.2	15,15	6,9.3	10,11	22,25	13,14	15,20	13,16	X,X	19,20	NA^‡^	NA^‡^	NA^‡^	NA^‡^	NA^‡^	32,33.2	12,13	11,12	11,14	10,11	22,25	13,16	X,X	19,20	8,11	12,13	NA^‡^	NA^‡^	NA^‡^

*Loci duplicated in different kits

†Consensus profile.

‡Not contained in the kit used.

§Missing data.

IIFalse homozygous locus.

**Table 4a:** Evaluation of autosomal short tandem repeat (STR) typing of Sample 1 by the laboratories participating in Collaborative Exercise*

Laboratory code	Number of loci typed (depending on the kit used)	Number of loci with concordant results	Missing one allele at heterozygous locus	Number of loci with wrong results	Number of loci with no results	Percentage of success
2	19	5	2	1	11	36.8
3	26	8	7	1	10	57.7
4	15	2	1	2	10	20.0
5	23	19	4	0	0	100.0
8	17	11	2	0	4	76.5
9	20	2	4	2	12	30.0
10	19	1	0	0	18	5.3
12	20	13	0	0	7	65.0
13	18	12	0	4	2	66.7
14	21	12	4	1	4	76.2
15	22	1	0	0	21	4.5
16	18	2	1	0	15	16.7
17	9	9	0	0	0	100.0
18	9	9	0	0	0	100.0

*Loci duplicated in different kits are counted twice, including AMELOGENIN. Laboratories 3 and 13 encountered a problem with pull-up peaks in STR loci vWA (laboratory 3) and D21S11, D18S51, and vWA (laboratory 13). A locus was considered correct if concordant with organizer results (or consensus profile) or if one of the two alleles at a heterozygous locus was detected. The calculations of success rate (%) are based on a total of the loci used by the laboratory and readers should take into consideration that the number of STRs ranged from 9 to 26.

**Table 4b:** Evaluation of autosomal short tandem repeat (STR) typing of Sample 2 by the laboratories participating in Collaborative Exercise

Laboratory code*	Number of loci typed	Number of loci with correct results	Missing allele in heterozygous locus	Number of loci with wrong results	Number of loci with no results	Percentage of success
2	30	30	0	0	0	100
3	17	17	0	0	0	100
4	17	17	0	0	0	100
5	23	23	0	0	0	100
7	27	27	0	0	0	100
8	17	17	0	0	0	100
9	22	15	2	0	5	77.3
10	18	18	0	0	0	100
12	22	21	1	0	0	100
13	17	17	0	0	0	100
14	22	22	0	0	0	100
15	24	24	0	0	0	100
16	21	21	0	0	0	100
17	22	22	0	0	0	100

*Laboratories 2-8, 10, and 13-17 provided complete profile results with no discordance. Laboratory 9 did not obtain results for the loci D21S11, D2S1338, and FGA and failed to identify 1 of the alleles at the vWA and D18S51 loci using Identifiler Plus kit (Life Technologies, USA) but obtained correct results for the D2S1338 and D18S51 loci using the MiniFiler kit (Life Technologies, USA). Laboratory 12 failed to identify 1 of the alleles in the D16S539 locus using the Identifiler kit (Life Technologies, USA), but obtained correct results using the MiniFiler kit (Life Technologies, USA). A locus was considered a success if concordant with organizer results (or consensus profile) or if one of the two alleles at a heterozygous locus was detected.

Only two laboratories provided results for X-STR loci for Sample 2 and both obtained the same results as the organizing laboratory (Table 5). No laboratory submitted X-STR results for Sample 1.

**Table 5:** Results of X-chromosomal short tandem repeat (STR) typing of Sample 2 by the laboratories participating in Collaborative Exercise

Laboratory code	X-STRs												
AME	DXS7132	DXS7423	DXS8378	DXS10074	DXS10079	DXS10101	DXS10103	DXS10134	DXS10135	DXS10146	DXS10148	HPRTB
1	X,X	12,13	14,14	10,12	14,17	19,21	28.2,32	17,18	36,41.3	25,32	30,40.2	27.1,27.1	12,14
3	X,X	12,13	14,14	10,12	14,17	19,21	28.2,32	17,18	36,41.3	25,32	30,40.2	27.1,27.1	12,14
7	X,X	12,13	14,14	10,12	14,17	19,21	28.2,32	17,18	36,41.3	25,32	30,40.2	27.1,27.1	12,14

### mtDNA typing

Four laboratories submitted mtDNA typing results for Sample 1, and 6 laboratories submitted results for Sample 2 (Table 6). Laboratories used different protocols for mtDNA amplification. Laboratories 1, 3, and 19 used primers as described by Eichmann et al (22), Laboratory 4 used primers that generated amplicon sizes of 385 bp (HVR1) and 240 bp (HVR2), Laboratory 6 used primers that generated amplicon sizes of 220 bp (HVR1) and 242 bp (HVR2) (23,24), Laboratory 11 used primers that generated amplicon sizes of 461 bp (HVR1) and 445 bp (HVR2), Laboratory 13 used primers that generated amplicon sizes of 449 bp (HVR1) and 391bp (HVR2), and Laboratory 15 used primers that generated amplicon sizes of 249 and 228 bp (HVR1), and 203 and 301 bp (HVR2).

**Table 6:** Results of mitochondrial DNA (mtDNA) typing performed by laboratories participating in the Collaborative Exercise

Laboratory code	Reported haplotype*	
Sample 1		
	HVR1 (range of sequencing)	HVR2 (range of sequencing)
1	16293A/G 16311C [15989-052]	195C, 263G [001-293, 317-460]
3	16293A/G 16311C/T 16362C/T [15989-052]	195C 263G [16533-619]
4	16104T 16126C 16294T 16304C [16050-16400]	73G [072-240]
11	16093C 16189C 16270T [16000-16461]	073G 146C 150T 263G [034-479]
15	16293G 16311C [15990-16239; 16163-16391]	195C [48-251; 164-465]
Sample 2		
	HVR1	HVR2
1	16304C 16311C [15975-042]	152C 263G [16524-635]
3	16304C 16311C [15989-052]	152C 263G [16533-619]
6	16304C 16311C [16128-16348]	152C 263G [45-287]
11	16304C 16311C [16000-16461]	152C 263G [034-479]
13	16304C 16311C [15978-16427]	152C 263G [9-399]
15	16304C 16311C [15990-16239; 16163-16391]	152C 263G [48-251; 164-465]
19	16304C 16311C [15989-052]	152C 263G [16533-619]

*The haplotypes obtained for the bone samples from the participating laboratories. Laboratory 3 reported problems with the read of the Sample 2 HVR2 sequence. Laboratory 6 reported interpretation difficulties for Sample 1 and therefore did not report the sequence data. Sample 2 provided consistent mtDNA results among the submitting laboratories; homopolymer stretches were not included in the comparison as these subregions are often not used in forensic analyses and interpretation varies among laboratories (21). There were a number of inconsistencies among the laboratories regarding mtDNA results for Sample 1. No consensus approach could be achieved with the data from Sample 1.

## DISCUSSION

### DNA quantitation and STR typing

The majority of participating laboratories quantified DNA extracts before performing PCR. The substantial differences that were obtained in DNA quantitation results could have resulted from the extraction efficiency (ie, chemistry), quantitation methodology (eg, using Qubit, which is not human specific, fluorometric vs real-time quantitative PCR, or using a mtDNA specific vs nuclear specific assay), and different final elution volumes. Whether these differences had any effect on the typing success was not considered, as the focus of this particular CE was to assess the typing success irrespective of the methodology.

DNA typing of Sample 2 was 100% complete and concordant by 13 of 14 laboratories. Thus, the STR typing procedures for forensic bone samples (6) yielded consistent results with a number of variations in extraction and amplification chemistries. The majority of laboratories used a silica-based extraction methodology, and although sample sizes were small, those laboratories tended to achieve a higher number of correctly called STR loci for the difficult Sample 1. In contrast, laboratories that used phenol/chloroform extraction chemistry tended to achieve a lower success in typing results. Similar observations in performance between phenol/chloroform and silica based extraction methodologies were described for bone samples from mass graves in the former Yugoslavia (13). The correlation of STR typing kits and the DNA typing success rates for difficult Sample 1 slightly favored use of a combination of MiniFiler and Identifiler Plus kits) (Life Technologies, USA).

The CE organizers did not ask the participating laboratories to provide the thresholds and interpretation guidelines to keep the format manageable for this first exercise. It is reasonable to assume that some success differences among laboratories may be due to interpretation and not solely to methodology and amplicon size. The next round of CE on bones should collect this additional information.

### mtDNA Typing

Sample 1 results differed among all 4 laboratories and the organizing laboratory. Sample 2 contained a sufficient amount of relatively intact DNA and all 6 submitting laboratories reported a haplotype concordant with that of the organizing laboratory, when omitting the homopolymeric C-stretch results in HVR2. Due to heteroplasmy and interpretation difficulties at this homopolymer region, results are rarely used in forensic analyses and interpretation of such results can vary (25). Compared with the success rate of mtDNA typing with other mtDNA collaborative exercises (26-31), the success rate in this CE was much lower for Sample 1 but comparable for Sample 2. However, the lower success for Sample 1 was to be expected. This sample was several hundred years old and highly degraded, as opposed to samples from other mtDNA CEs that were typically of higher quality, such as hairs and stains. Relatively few laboratories submitted the results of mtDNA typing (4 laboratories for Sample 1, 6 laboratories for Sample 2), but the number of different mtDNA profiles reported for Sample 1 suggests that mtDNA typing in challenging and degraded bone samples is not a robust and reliable methodology for some laboratories and more investigation is needed. One possible explanation for the variable mtDNA sequence results may be that the primers used by the participating laboratories generate amplicon sizes that are larger than the fragmented DNA in Sample 1 and, thus, may select for low level contaminating exogenous DNA.

### The concept of CE

The selection of a femur for the samples was based on the greater amount of material that could be obtained for distribution among laboratories. Recent findings might suggest that a femur may not be the best choice (32), but the amount of available specimen must be considered when preparing a sufficient quantity of operationally identical samples for all participating laboratories. The concept of future CEs on bone samples could clarify the typing results from the extraction-borne variations by sending the participating laboratories DNA extracted from aged bone samples. This approach may help to better identify the root cause(s) of particular DNA typing limitations, eg, the extraction method. The next CE could also address the cleaning and grinding phase and the removal of humic acid inhibitors and modern DNA contaminants (18,33-41). Another improvement of the CE concept would be the inclusion of massive parallel sequencing (42,43) during the verification of the sample by the organizing laboratory.

### C**onclusion and methodology recommendations**

The analysis of highly degraded and difficult bone samples, such as archeological specimens, may not yield reliable results in all laboratories. Contamination may be a concern that should be investigated further. Perhaps development of a quality-controlled commercial kit could reduce some forms of contamination. Those laboratories with inconsistent results may consider that findings should not be reported unless they are confirmed by a second independent laboratory (44). Future collaborative exercises could include male skeletal remains and Y-chromosomal STR typing to further investigate performance among laboratories.

The first recommendation we may make regarding the methodology is that DNA analysis of human skeletal remains should adhere to ethical and data protection issues. Furthermore, laboratories should establish procedures for efficient reduction of possible sources of contamination, such as separated bone extraction area, forensic grade consumables, and cleaning of the laboratory areas. Laboratories should use an extraction procedure providing the highest DNA yield and purity, eg, possibly silica-based extraction chemistry. Laboratories should determine the quantity of extracted DNA using a human mtDNA specific quantitative real-time PCR assay that also includes at least two internal positive controls to evaluate the presence of inhibitors and degradation. Laboratories should include an additional step of PCR inhibitor removal for samples with detected inhibition. Last but not least, laboratories should use short amplicons for both STR and mtDNA typing for analysis of very challenging samples.
